# A Fully Integrated Microfluidic Device with Immobilized Dyes for Simultaneous Detection of Cell-Free DNA and Histones from Plasma Using Dehydrated Agarose Gates

**DOI:** 10.3390/gels10030186

**Published:** 2024-03-08

**Authors:** Shadi Shahriari, P. Ravi Selvaganapathy

**Affiliations:** 1 Department of Mechanical Engineering, McMaster University, Hamilton, ON L8S 4L8, Canada; shahris@mcmaster.ca; 2 School of Biomedical Engineering, McMaster University, Hamilton, ON L8S 4L8, Canada

**Keywords:** sepsis, microfluidics, cfDNA, histones, xurography, agarose

## Abstract

Sepsis, a life-threatening condition resulting from a failing host response to infection, causes millions of deaths annually, necessitating rapid and simple prognostic assessments. A variety of genomic and proteomic biomarkers have been developed for sepsis. For example, it has been shown that the level of plasma cell-free DNA (cfDNA) and circulating histones increases considerably during sepsis, and they are linked with sepsis severity and mortality. Developing a diagnostic tool that is capable of assessing such diverse biomarkers is challenging as the detection methodology is quite different for each. Here, a fully integrated microfluidic device capable of detecting a genomic biomarker (cfDNA) and a proteomic biomarker (total circulating histones) using a common detection platform has been demonstrated. The microfluidic device utilizes dehydrated agarose gates loaded with pH-specific agarose to electrophoretically trap cfDNA and histones at their respective isoelectric points. It also incorporates fluorescent dyes within the device, eliminating the need for off-chip sample preparation and allowing the direct testing of plasma samples without the need for labeling DNA and histones with fluorescent dyes beforehand. Xurography, which is a low-cost and rapid method for fabrication of microfluidics, is used in all the fabrication steps. Experimental results demonstrate the effective accumulation and separation of cfDNA and histones in the agarose gates in a total processing time of 20 min, employing 10 and 30 Volts for cfDNA and histone accumulation and detection, respectively. The device can potentially be used to distinguish between the survivors and non-survivors of sepsis. The integration of the detection of both biomarkers into a single device and dye immobilization enhances its clinical utility for rapid point-of-care assessment of sepsis prognosis.

## 1. Introduction

Sepsis arises from abnormal host response to an infection which leads to critical organ dysfunction [1]. It is estimated that there are about 31.5 million sepsis cases with 5.3 million deaths worldwide each year [2]. In septic patients, the risk of mortality increases by 8% for every hour of delay in sepsis treatment [3]. Identifying severe sepsis early on could result in timely interventions that have the potential to decrease sepsis mortality. Therefore, the clinical utility of prognostic and diagnostic biomarkers in sepsis is significant [4]. Many biomarkers have been studied for their use in sepsis prognosis. Notably, procalcitonin (PCT) and C-reactive protein (CRP) have been extensively considered biomarkers for the diagnosis and prognosis of sepsis [5]. More recently, cell-free DNA (cfDNA) and histones have been introduced as two biomarkers for sepsis prognosis which both reflects cellular death activity [2,6,7,8,9,10]. Nucleosomes consist of 145 bp DNA wrapped around a core histone octamer, which contains two copies of histones H2A, H2B, H3, and H4. Widespread cell death during sepsis can contribute to the extracellular release of DNA and histones. Circulating levels of nucleosomes, cfDNA, and histones are increased in sepsis [11,12,13,14]. Neutrophils are the major source of released cfDNA. High concentrations of cfDNA showed high discriminative power for prediction mortality among patients with severe sepsis [15]. A study showed that the mean cfDNA level in sepsis survivors was 1.16 ± 0.13 µg/mL, which is similar to that of healthy persons (0.93 ± 0.76 µg/mL); however, for non-survivors, the level was 4.65 ± 0.48 µg/mL [7]. 

Similarly, as the result of the rupture of the nuclear and plasma cell membrane which happens during organ injury, histones are released into extracellular environment. Studies showed that high levels of circulating histones are significantly linked with sepsis severity and mortality. Free histones have not been found in healthy persons or detected in very low concentrations [6,10]. Histones are basic proteins due to their N-terminal tail rich in lysine and arginine, which forms the basic nature of the histones [2]. Histones have an isoelectric point around 11 [16]. A study showed that total free histone concentration in sepsis patients who died within 28 days of ICU admission was 32.7 µg/mL; however, for survivors, this was 20.1 µg/mL. Total circulating histone level for healthy persons was 1.3 µg/mL [6]. 

Detecting genomic and proteomic biomarkers simultaneously can be challenging due to inherent differences in the nature of genetic material (genomic) and proteins (proteomic). Therefore, having a rapid and simultaneous method for detection of cfDNA (genomic biomarker) and circulating histones (proteomic biomarker) can be beneficial by offering a better understanding of the sepsis progression and aid physicians in prognosis. One of the methods that has been extensively used for the detection and separation of biomolecules in microfluidics is gel electrophoresis [17]. 

Previously, we showed microfluidic devices containing dried agarose gels inside membranes for concentration and detection of biomolecules [18,19]. The dried gels can be reconstituted in the sample solution itself and function effectively, thereby eliminating the need to load the gels into devices at the time of testing making this approach suitable for point of care applications. These gels can be used in a variety of ways to either serve as a entangling and concentration medium for DNA or as gels loaded with specific pH to trap biomolecules at their isoelectric point [18,19]. Nevertheless, these devices still required the premixing of fluorescent dyes with the sample solution prior to the introduction into the device, which is not suitable for point of care testing. Also, the dyes required for genomic and proteomic biomarkers are markedly different, and the integration of these dyes into the device itself and ensuring minimal cross reactivity can greatly facilitate their use in the field. 

Here, we introduce the design of a single microfluidic device with two dehydrated agarose gates and incorporated fluorescent dyes for the accumulation and detection of histones and cfDNA from the plasma sample in 20 min total. This device can be enhanced further as a point of care (POC) device for the rapid quantification of histones and cfDNA as a prognostic biomarker in plasma of individuals with sepsis.

## 2. Results and Discussion

### 2.1. Device Design and Working Principle

The goal of the device is to detect both cfDNA and total circulating histones in the plasma of sepsis patients to quickly distinguish between survivors and non-survivors. This can be accomplished by tagging DNA and histones with different fluorescent dyes and isolating and accumulating each of them in agarose gel membranes embedded inside a microfluidic device. The fluorescent dyes are immobilized inside the sample reservoirs of the device which eliminates the need for premixing the sample and dye outside the device. This was performed by depositing and drying the dyes on the surface of a PET film in the location of sample reservoirs and positioning the film under the sample channel. The agarose gates are dehydrated agarose gels loaded onto porous PTFE membrane with pH 8.5 and pH 11 for cfDNA and histone trapping, respectively. The porous PTFE membranes are beneficial for incorporating hydrogels during the device fabrication process. These membranes help preserve the structure of dehydrated hydrogels and facilitate rehydration as required [18]. 

**Figure 1:**
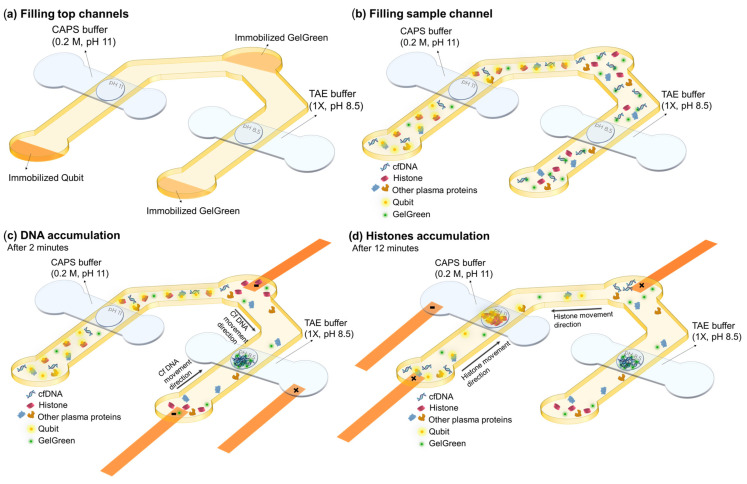
Working principle and steps of the device. (**a**) Filling the top channels with the corresponding buffers for agarose rehydration. (**b**) Filling the sample channels and waiting for labelling DNA and proteins with their corresponding dyes. (**c**) DNA accumulation by applying 10 V to the channels connected to agarose gate with pH 8.5. (**d**) Histone separation and detection by applying 30 V to the channels connected to the agarose gate with pH 11.

The microfluidic device was made from pressure sensitive adhesives and films using xurography. Xurography has been used in all the fabrication steps from agarose integration to dye immobilization and device fabrication. Xurography is a rapid and low cost method for the fabrication of microfluidic devices by cutting and attaching different adhesives and polymer films [20]. The device consists of six layers which form four channels (two channels on top and two channels with one inlet in bottom) with a sandwiched layer containing dried agarose in between and a layer containing dried fluorescent dyes in the back (Figure 1). A stack of four layers of hydrophilic porous PTFE membrane (36 μm thick each) was used as the scaffold for integration of agarose. These PTFE layers were sandwiched between two double sided adhesives, Kapton tape, and a silicone-based adhesive (AR-94119). AR-94119 showed minimal auto-fluorescence with little non-specific binding of dye or labeled proteins to its surface. These two double sided adhesives have circular openings (1 mm in diameter) for filling the exposed PTFE membranes with agarose in those regions to form gel gates. Agarose solutions (3% *w*/*v*) made in CAPS buffer with pH 11 and TAE buffer with pH 8.5 were loaded in the open parts of porous membrane to create the gates for histones and DNA, respectively. Qubit protein assay dye was used for labelling proteins and GelGreen for staining DNA. The fluorescent dyes were patterned on the surface of a hydrophilic PET film (3M-9984) using a screen made of Kapton tape with three rectangles cut-outs attached to the surface of the PET film for dye loading. After drying the dyes on the film in a desiccator, Kapton tape is peeled off, leaving patterned dried dyes at appropriate locations on the PET film. Top channels (buffer channels) layer was out of PVC film, and it was attached to the sandwiched layer containing agarose gates from the Kapton tape side. The top channels were sealed with a hydrophilic single-sided adhesive (AR-93049) containing the inlets. The layer containing the bottom channels was cut out of Kapton tape and was attached to the sandwiched layer from silicone-based adhesive (AR-94119) side. Then, the electrodes were positioned at the two ends of bottom channels and one end of top channels. Finally, the device was sealed with the PET film layer containing immobilized dyes. 

At the time of experiment, the top channels placed over agarose with pH 8.5 (made in TAE) and agarose with pH 11 (made in CAPS) gates were filled with 1X TAE and 0.2 M CAPS buffer with the same pH of the agarose gates, respectively (Figure 1a). After agarose rehydration with their corresponding buffer, the bottom channels were filled with the sample containing DNA and histones. The sample was pipetted several times in each of the reservoirs for better mixing the sample and the dried immobilized dyes. Waiting times of about 2 min and 12 min were needed for DNA/GelGreen and proteins/Qubit labelling, respectively (Figure 1b). First, the objective was centered on the agarose membrane for DNA detection. After 2 min, 10 V was applied between the two ends of bottom channel under the agarose gate (pH 8.5) for DNA accumulation and top channel containing TAE buffer. Fluorescent images from the agarose membrane were taken every 30 s for a total of 5 min using a raspberry pi camera. By applying electric field, DNA tagged with GelGreen moved from bottom channel towards the positive electrode in buffer channel due to its negative charge. The DNA is accumulated in agarose gel inside the membrane as it provides a lower mobility environment (Figure 1c). Based on the fluorescent intensity captured from the agarose gate after 5 min, the DNA concentration can be quantified for different samples. 

After 12 min, the objective was centered on the other agarose gate (pH 11) and the filter cube was changed to the one matching Qubit dye. Electric field (30 V) was applied between the bottom channel under the agarose gate and buffer channel filled with CAPS buffer (Figure 1d). The direction of the electric field was switched as the electrodes in sample channel were positive and the one in top channel was negative. Fluorescent images of the agarose gate were taken every 30 s for a total of 5 min using a raspberry pi camera. The device design was intended to prevent the entry of the majority of plasma proteins into the agarose membrane with pH 11, while histones become trapped in the isoelectric membrane. All the proteins were labeled with Qubit dye. Blood plasma has pH around 7.4. Therefore, pH 7.4 serves as a cut-off point for determining the charge of proteins and their direction of movement in the sample channel. In the presence of electric field, proteins with an isoelectric point lower than the sample pH acquire a negative charge and migrate towards the positive electrodes located in the reservoirs of the sample channel, consequently moving away from the agarose gate. However, proteins with an isoelectric point greater than that of the sample, such as histones, acquire positive charge, leading them to move toward the cathode in the upper channel. As histones enter the agarose gel membrane with a pH of 11, their charge diminishes, causing them to slow down and accumulate. Consequently, the quantification of histones is possible by assessing the fluorescent intensity in the agarose gate. As most plasma proteins are acidic, they migrate towards the positive electrodes in the sample channels, and those with a pI higher than 7.4, which are much less abundant, migrate towards the agarose membrane. Hence, a significant portion of proteins is excluded from entering the gate. The proteins with a pI greater than 7.4 either pass through the gate or become trapped in the membrane, depending on their pI values. 

### 2.2. Tagging and Accumulating Histones and DNA in the Devices with Immobilized Dye

**Figure 2:**
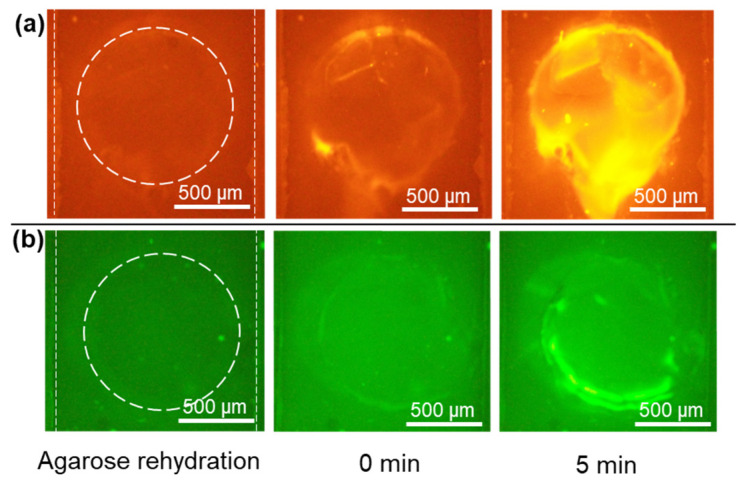
Tagging and concentration of (**a**) histones and (**b**) 150 bp DNA in the devices with integrated dried fluorescent dyes.

To demonstrate the possibility of immobilizing Qubit and GelGreen in a dried state inside the device and still efficiently tagging and accumulating proteins and DNA, experiments using two samples of histones and DNA in buffer were performed. First, two devices with two perpendicularly overlaid channels, one containing agarose in TAE buffer (pH 8.5) with immobilized GelGreen in sample reservoirs (5X diluted) and another one with agarose in CAPS buffer (pH 11) and immobilized Qubit in reservoirs, were fabricated. The devices were tested the next day. An amount of 1 mg/mL of calf thymus histones was diluted from the stock solution in 50 mM Tris-HCl buffer, pH 8. For testing the device with immobilized Qubit, at the time of experiment for agarose rehydration, the top channel was filled with CAPS buffer with pH 11 similar to the pH of agarose isoelectric gate. An image was taken from the agarose gate after the rehydration (Figure 2a). As a result of agarose rehydration, the membrane section turned transparent. Then, the bottom channel was filled with 1 mg/mL of histones in the buffer sample. The sample was pipetted several times into the sample inlet for enhancing the mixing between the sample and immobilized Qubit. After 5 min of waiting for the binding of the Qubit and histones, 30 V was applied between the channels. When the electric field was applied, histones tagged with Qubit, possessing a positive charge in the sample solution (pH 8), started to move toward the cathode and the agarose gate. Upon reaching the agarose gate with pH of 11, histones largely lost their charge and became trapped. Figure 2a shows the change in fluorescence intensity in the gate at the time of applying voltage and after 5 min. At 0 min, there was a slight fluorescence increase from filling the channel with sample and mixing it with Qubit. The fluorescent intensity started to increase when applying the electric field. 

A similar experiment was designed for the accumulation of DNA in a device with immobilized GelGreen. After filling the top channel with 1X TAE buffer for agarose rehydration, the bottom channel was filled with 5 µg/mL of 150 bp DNA diluted from stock solution in the 50 mM Tris-HCl buffer. The sample was pipetted several times into the sample channel reservoirs for the better mixing of dried GelGreen and DNA. After 2 min of waiting, 10 V was applied. By applying the electric field, the DNA due to its negative charge migrated towards the anode in the buffer channel and was trapped inside the agarose-loaded membrane as the agarose mobility was lower. Figure 2b shows the change in fluorescence intensity in the membrane at the time of applying voltage and after 5 min. At 0 min, there was a small fluorescence increase from filling the channel with DNA sample and its binding with GelGreen. The fluorescent intensity started to increase after applying the electric field. 

Therefore, it was shown that both Qubit and GelGreen can be immobilized and stored inside the microfluidic device, eliminating the need for premixing the sample and dye outside the device. Both histones and DNA can be tagged efficiently with dried Qubit and GelGreen, respectively, and accumulate inside the rehydrated agarose. However, the sample and dye still need to be mixed inside the device to obtain a homogeneous mixture of biomolecules labelled with their respective dyes. A failure to mix in the reservoir can result in the uneven distribution of substances and suboptimal biomolecules labeling, leading to a poor signal. To enhance mixing in the future, several improvements such as incorporating mixing elements and electrokinetic mixing could be considered. 

### 2.3. Trapping Histones in the Presence of High Concentration of Other Background Proteins (BSA) in a Device with Immobilized Qubit

The capability of the device with immobilized Qubit to detect histones in the presence of other proteins, such as a high concentration of albumin, was examined. Samples were prepared by mixing 40 mg/mL of BSA with different concentrations of histone. BSA was chosen due to its similarities in properties such as isoelectric point and weight to human serum albumin (HSA). Albumin constitutes a significant portion of blood plasma proteins, with a concentration ranging from 35 to 50 mg/mL and an isoelectric point around 5. Consequently, BSA with concentration of 40 mg/mL was chosen to mimic albumin in plasma. Considering the isoelectric point of BSA to be around 5 and histones around 11, they acquire opposite charges in the sample solution with pH of 8. BSA acquires a negative charge, while histones obtain a positive charge in the solution. Thus, when subjected to an electric field, they migrate in different directions. 

**Figure 3:**
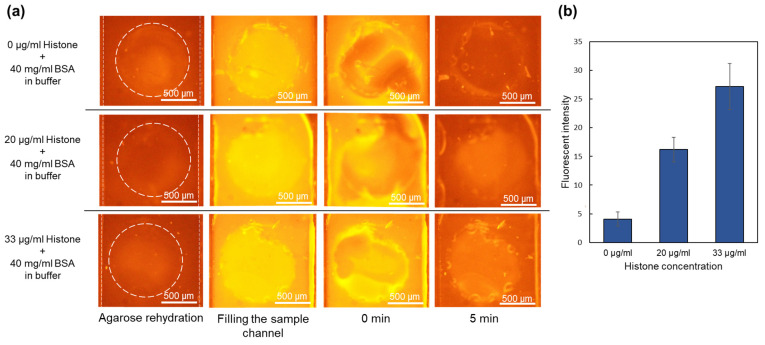
Trapping histones in the presence of 40 mg/mL BSA in the devices with immobilized Qubit. (**a**) Images captured at the time of filling the sample channel, at the time of applying voltage (0 min), and after 6 min for different samples. (**b**) Fluorescence intensity for each concentration of histone in the sample containing BSA (N = 3).

A similar device with 2 μL of one-time diluted Qubit dried in the sample channels reservoirs was used. A new device was used for each repeat of the experiment. After filling the top channel with CAPS buffer, the bottom channel was filled with the sample containing 40 mg/mL BSA and 0, 20, and 33 µg/mL of histone. The sample was mixed several times with dried Qubit inside the reservoirs by repeated pipetting. After 5 min of waiting time for the binding of the proteins and Qubit, 30 V was applied between the channels. Images are shown in Figure 3a for the sample containing only BSA and BSA mixed with 20 and 33 µg/mL of histones at 0 and 5 min. In all the cases, the bottom channel shows a high fluorescence intensity after filling and mixing the sample in the channel. This is because of the high concentration of BSA (40 mg/mL). After the waiting time for the complete binding of the proteins and Qubit dye, the electric field was applied. In a pH 8 environment, BSA gains negative charge and moves away from the agarose membrane toward the positive electrodes at the ends of the sample channel. Meanwhile, histones move toward the gate and become trapped. The average intensity in the gate after 5 min for samples with 0, 20, and 33 μg/mL of histones is plotted in Figure 3b. As depicted in the images, after 5 min, the fluorescence intensity is highest in the gate for the 33 µg/mL sample. The intensity is reduced with a decreasing concentration of histones in the sample. In the control sample, nearly all of the BSA migrated toward the electrodes, and the membrane region was almost cleared of BSA after applying an electric field for 5 min. This indicates that the device with immobilized dried Qubit effectively distinguishes between various levels of histones even in the presence of a high amount of albumin.

### 2.4. Accumulation of DNA in the Presence of Histone in a Device with Immobilized GelGreen Dye

**Figure 4:**
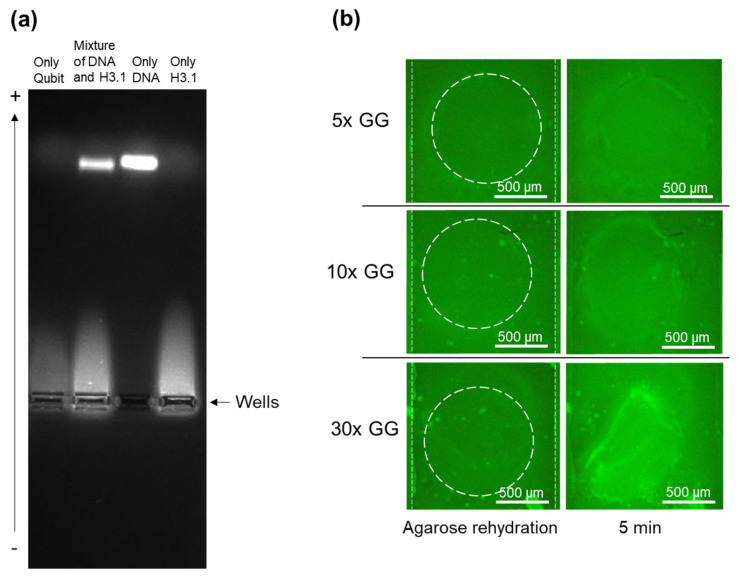
Accumulation of DNA in the presence of histones. (**a**) Gel electrophoresis of DNA, histone, and mixture of DNA and histone. (**b**) Tagging and separating DNA in the presence of histone with different dilution ratios for immobilized GelGreen.

In the next step, we studied the interference in DNA accumulation by presence of histones in a sample containing both. The DNA binds to histone due to their natural functions and charges. In order to identify whether free DNA is present in a mixture sample, a gel electrophoresis was performed. Four samples comprising one of only Qubit; one with a mixture of 150 bp DNA tagged with GelGreen and histones tagged with Qubit with the similar weight ratio between the cfDNA and histones in the case of non-survivors; one with only 150 bp DNA tagged with GelGreen; and one with only histone tagged with Qubit were created. These samples were loaded into four wells of a 2% agarose gel, and electrophoresis was performed at 75 mA for 45 min. As is shown in Figure 4a, a DNA band still formed in the sample containing both DNA and histones; however, the band was smaller compared to its corresponding DNA weight in the sample containing only 150 bp DNA. This shows that only a small portion of DNA in the mixture sample was affected by the electrostatic interaction between DNA and histones, and the majority of the DNA or DNA-bound histones remained negatively charged. This shows that the designed device can be used for accumulating DNA and histones from a mixed sample in agarose gates with the aforementioned working conditions.

We also tested this separation in the device containing immobilized GelGreen to investigate the functionality of the device with immobilized dye for the samples containing both DNA and histones. However, the 5X diluted GelGreen that was used previously did not provide enough dye for the mixture of 150 bp DNA and histones. Therefore, less diluted immobilized GelGreen was used. Devices with 2 µL of 10X and 30X GelGreen deposited in the reservoirs were fabricated. The bottom channel was filled with the sample containing 5 µg/mL of 150 bp DNA and 30 µg/mL of histone. After waiting for the staining of the DNA with GelGreen, 10 V was applied between channels. Following 5 min of applying voltage, devices with 30X GelGreen displayed enough dye for the tagging of 5 µg/mL of 150 bp DNA in the presence of 30 µg/mL of histones (Figure 4b). This dilution ratio was selected for the rest of the experiments.

### 2.5. Accumulation of DNA Spiked in Plasma in the Presence of Histone in the Device with Immobilized GelGreen

**Figure 5:**
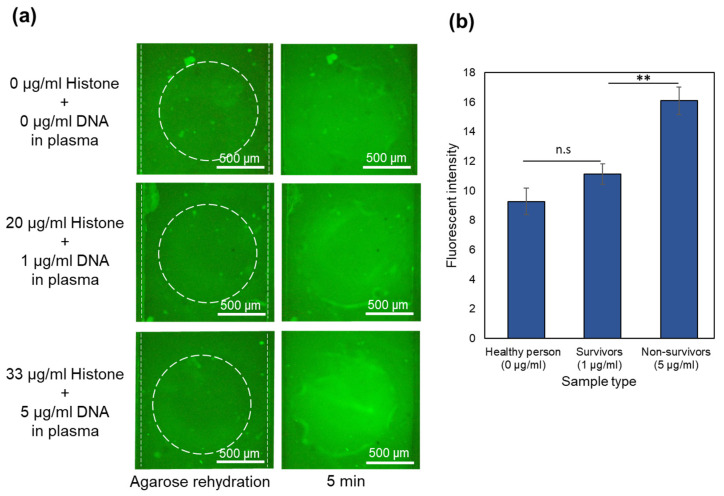
Accumulation of 150 bp DNA spiked in plasma in the presence of histone in the devices with 30X GelGreen. (**a**) Images captured after agarose rehydration and after 5 min of applying 10 V for samples of healthy persons, survivors, and non-survivors. (**b**) Fluorescence intensity values for DNA concentrations corresponding to survivors and non-survivors (N = 3). Statistical significance for samples were calculated by the two-tailed *t*-test (n.s: not significant, ** *p* < 0.01).

After testing the device with immobilized GelGreen with mixture of DNA and histones in buffer, devices with two regions of immobilized 30X GelGreen were created for the accumulation of 150 bp DNA spiked in plasma in the presence of histones. Three samples with the corresponding concentrations of cfDNA and histones in plasma for healthy persons (no spiked DNA or histones), survivors of sepsis (1 µg/mL 150 bp DNA and 20 µg/mL of histones), and non-survivors of sepsis (5 µg/mL 150 bp DNA and 33 µg/mL histones) were created. After the rehydration of agarose by TAE buffer in top channel and the filling of the sample channel, we waited for 2 min. Then, 10 V was applied to initiate the migration of DNA towards the agarose gate. As is shown in Figure 5a, after 5 min, the fluorescent intensity in the agarose gate was higher for the sample containing 5 µg/mL 150 bp DNA compared to the sample with 1 µg/mL of DNA. Therefore, using the device with immobilized 30X GelGreen for tagging the cfDNA, the survivors and non-survivors of sepsis can be differentiated by the fluorescent intensity in the agarose membrane (Figure 5b).

### 2.6. cfDNA and Histone Detection from Human Plasma Samples in Fully Integrated Device

**Figure 6:**
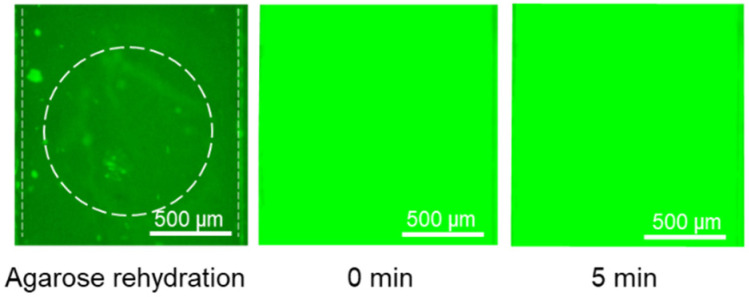
Fluorescence saturation inside the channel for DNA detection due to interference from plasma proteins tagged with Qubit when both dyes are mixed with the sample.

After demonstrating that 150 bp DNA and histones can be tagged with their dried immobilized fluorescent dyes and accumulate in their corresponding agarose gates separately, we showed the possibility of integrating both of these accumulations in the same device with a single inlet for the sample and two agarose gates for concentrating DNA and histones. One of the challenges for integrating detection of both in a single device was the overlapping between the excitation and emission wavelengths of GelGreen and Qubit, which can cause difficulty in fluorescent imaging if both are present in the sample at the same time. It was shown that if the plasma sample is mixed with both GelGreen and Qubit, the fluorescent intensity emitting from the DNA/GelGreen is blocked due to the high concentration of plasma proteins tagged with Qubit (Figure 6). Therefore, dyes need to be immobilized in different locations to minimize the chance of them mixing. Consequently, Qubit was deposited inside only one of the reservoirs of the sample channel under the agarose gate with pH 11. GelGreen was deposited in the main inlet as well as the other outlet of the sample channel separated from Qubit. With this deposition arrangement, Qubit is only mixed with the sample part connected to the pH 11 agarose gate for histone accumulation and would not affect the fluorescent signal from DNA/GelGreen in the other channel. It should be noted that in the case of histone accumulation, the movement of histones and DNA is in the opposite direction, and therefore, the presence of GelGreen in the channel for histone detection would not affect the final fluorescent signal from the agarose gate at a pH 11.

The final design for the integrated device consists of two agarose gates and immobilized fluorescent dyes with the optimized dilution ratio in separate reservoirs. The device is used for accumulating of both 150 bp DNA and histones from plasma. Three different samples resembling the plasma samples of healthy persons, survivors of sepsis, and non-survivors of sepsis were tested. To prepare the samples, a similar protocol to that previously mentioned was followed. The deposited Qubit was not diluted in this case as the total plasma protein level reached 80 mg/mL. Also, the 30X GelGreen was deposited in the sample main inlet and the outlet of the channel connected to agarose gate with pH 8.5 during device fabrication. The experimental setup and conditions were the same as before. After 2 min of waiting time for the staining of DNA and 5 min of applying the 10 V for DNA accumulation, the objective was centered on the other agarose gate for histone detection. The filter cube was also changed to the other one, suitable for Qubit. After 12 min of waiting for the tagging of proteins with Qubit, 30 V was applied for 5 min between the channels for histone accumulation. The experiment was repeated three times for each concentration with a new device used in each repeat.

**Figure 7:**
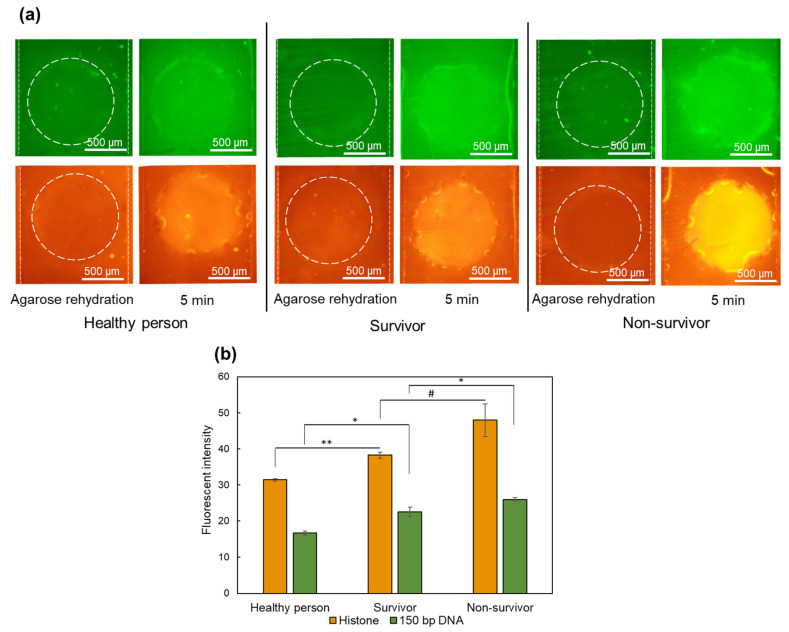
Trapping and concentrating 150 bp DNA and histones from spiked plasma samples in a fully integrated device. (**a**) Images captured after agarose rehydration and after 5 min of applying 10 V for DNA detection and 30 V for histone detection in the plasma samples of healthy persons, survivors, and non-survivors. (**b**) Plot showing changes in measured fluorescent intensity for DNA and histones for samples of healthy person, survivors, and non-survivors (N = 3). Statistical significance for samples calculated by the two-tailed *t*-test (** *p* < 0.01, * *p* < 0.05, # *p* < 0.1).

As it is shown in Figure 7a, the fluorescent intensity in the agarose gate for both 150 bp DNA and histones after 5 min of applying the electric field is higher for samples simulating the non-survivor condition compared to the survivor condition, and the levels for survivors are greater than those of a healthy person. Figure 7b also demonstrates the fluorescent intensity values for each of the biomarkers. This shows the device capability for differentiating between healthy persons, sepsis survivors, and sepsis non-survivors based on the concentrations of two biomarkers in less than 20 min using their plasma samples. In order to develop this device into a POC device for sepsis prognosis, additional characterization is needed regarding clinical patients’ blood samples. Calibration with a range of cfDNA concentrations is also necessary to establish device accuracy across different samples. Also, further modifications are required to enhance the device sensitivity and resolution, contributing to its effectiveness as a robust tool for sepsis prognosis.

## 3. Conclusions

This paper presents a rapid, low-cost, and fully integrated microfluidic device for the detection of cfDNA and free histones as prognostic biomarkers for sepsis. This microfluidic device detects both cfDNA and histones in a single platform by addressing previous limitations, such as the need for sample pretreatment and off-chip dye mixing. The study demonstrates the feasibility of immobilizing fluorescent dyes, Qubit for histones and GelGreen for DNA, in a dried state within the device while efficiently tagging and accumulating the respective biomolecules. The incorporation of dehydrated agarose gates and immobilized fluorescent dyes enhances efficiency and simplifies the process. Xurography, a rapid and cost-effective microfluidics fabrication technique, is employed for all device fabrication steps. The device was tested with 30 µL of samples containing both 150 bp DNA and histones spiked in plasma, representing healthy individuals, sepsis survivors, and sepsis non-survivors. Results demonstrated the device ability to differentiate between these groups based on the concentration of the two biomarkers within less than 20 min. This suggests the device potential as a point-of-care tool for sepsis prognosis, offering the rapid assessments of multiple biomarkers from patient plasma samples. However, more characterization is needed for using clinical patient samples, and the device needs to be calibrated with other concentrations. Additionally, for a point-of-care device, the DC power supply needs to be substituted with batteries. Moreover, simplifying the fluorescent microscope could be achieved by incorporating a LED light source and optical filters.

## 4. Materials and Methods

### 4.1. Materials

Histone H3.1 human, histones from calf thymus type II-A, and bovine serum albumin (BSA) were acquired from Millipore Sigma, Oakville, ON, Canada. NoLimits 150 bp DNA fragment, Qubit protein assay kit, Tris- HCl (1M solution and pH 8.0) and CAPS buffer (0.5 M, pH 11) were obtained from Thermo Fisher Scientific, ON, Canada. GelGreen nucleic acid gel stain, 10,000X in water, was obtained from Biotium, Fremont, CA, USA. Pooled human plasma K2 EDTA was purchased from Innovative research, Novi, MI, USA. CAPS buffer (0.2 M, pH 11) was obtained from Bioworld, Dublin, OH, USA. Agarose and 50x TAE buffer concentrate were purchased from BioShop Canada Inc, Burlington, ON, Canada. Hydrophilic PTFE unlaminated membrane (1 micron pore size) was acquired from Sterlitech, Auburn, WA, USA. Double-sided and double liner polyimide (Kapton) tape (100 μm thick) was purchased from Caplinq, Ottawa, ON, Canada. A double-sided silicone-based pressure sensitive adhesive (ARcare-94119, 142 µm thick) and one-sided hydrophilic acrylic-based adhesive (ARflow-93049, 100 μm thick) were obtained from adhesive research, Glen Rock, PA, USA. Surfactant-free and hydrophilic polyester film (3M-9984, 99 μm thick) was obtained from 3M™, Maplewood, MN, USA. Polyvinyl chloride (PVC) film (Clear-Lay, 127 μm thick) was obtained from Grafix, Maple Heights, OH, USA. Copper–polyimide composite foil (9 μm-Cu and 12 μm-PI thick) was obtained from DuPont, ON, Canada. 

### 4.2. Fabrication

The microfluidic device contains four channels, two agarose gates, and a layer containing dried fluorescent dyes. Two channels with a single inlet for the sample are present at the bottom and two channels are present at the top of each agarose gate for the rehydration buffer. Agarose is loaded in the membranes between bottom and top channels. The layer containing immobilized dyes is the bottommost layer. Xurography was used as the main fabrication method in all the steps. Cutting plotter (FC8600, Graphtec America, Irvine, CA, USA) with CB09UB blade was used for cutting different layers of the device. In the following, the process for agarose integration, dye immobilization, and device assembly is discussed in detail. 

#### 4.2.1. Integration of Agarose into the Device

**Figure 8:**
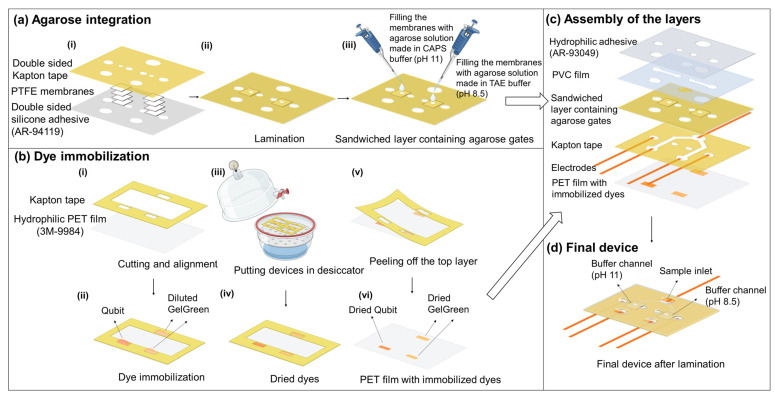
Fabrication steps of the final device. (**a**) Integration of agarose gates into the device using PTFE membranes and double-sided adhesives. (**b**) Steps for immobilization of the fluorescent dyes in the device. (**c**) Alignment and assembly of the layers. (**d**) Schematic view of the final device.

In order to integrate the two agarose gates into the device, first two layers of silicone-based double-sided adhesives (Kapton tape and AR-94119) were patterned using xurography based on the design for agarose gates (1 mm diameter circular openings) and device inlets. Then, four layers of hydrophilic PTFE membrane (each layer was 36 µm thick and 2.5 mm wide) were stacked and sandwiched between these two layers of double-sided adhesives under the circular openings (Figure 8a(i)). The layers were aligned and laminated (Figure 8a(ii)). Then, 3% agarose was made in two different buffers (0.5 M CAPS, pH 11, and 1X TAE buffer, pH 8.5). Each of the PTFE membrane circular openings was filled from both side of sandwiched layer with one of the agarose solutions made in CAPS and TAE buffer (Figure 8a(iii)). Following wicking the agarose solution into the membranes completely and its gelation, the excess amount of agarose was wiped off from the surface of the layer containing agarose, and the agarose gates were allowed to dry. 

#### 4.2.2. Immobilization of Fluorescent Dyes into the Device

GelGreen and Qubit were immobilized inside the microfluidic device by patterning and drying them on the surface of the bottom most layer of the device (hydrophilic PET film 3M-9984). To create the patterns of the dyes on the surface of the PET film, a layer of Kapton tape containing the cut-out patterns for dyes was used as a masking layer. The cut-out patterns were three rectangles with a width of 4.5 mm, of which two had a height of 1.8 mm and one had a height of 2.3 mm. This Kapton tape layer was attached to the PET film surface (Figure 8b(i)). Then, 2.5 µL of Qubit was pipetted into one of the smaller rectangular reservoirs and 2.5 µL of diluted GelGreen was pipetted into each of the other reservoirs (Figure 8b(ii)). Next, this layer containing the patterned dyes was put in a desiccator for assisting the evaporation of the dye solvent (Figure 8b(iii)). Afterwards, the dyes were dried completely in their locations (Figure 8b(iv)) and the top Kapton tape layer was peeled off from the PET film (Figure 8b(v)), leaving behind the patterned dried dyes on the film (Figure 8b(vi)). 

#### 4.2.3. Assembly of the Final Device

The rest of the layers of the microfluidic device were cut using xurography as well. Top channels of 1.5 mm width and 4.15 mm height were cut out of PVC film (127 μm thick). This layer was aligned and attached to the layer containing dried agarose gates from the Kapton tape side. The bottom channels with a single inlet and 1.5 mm width were cut from double-sided Kapton tape with one liner removed (170 μm thick), and it was attached to the sandwiched-dried agarose layer from the AR-94119 adhesive side. Then, the channels were sealed from the top using a hydrophilic adhesive (AR-93049) with the inlets of the channels. The electrodes of 1.25 mm width were cut out of the copper foil. They were placed in the reservoirs of the bottom channels and one of the reservoirs in the top channels. Then, the hydrophilic PET film (3M-9984) containing the dried dyes, was attached to the bottom channels layer and electrodes. The arrangement of the layers of the device is shown in Figure 8c. After the alignment and assembly of the layers, the device passed through a lamination step for a good sealing. The schematic of the final device is provided in Figure 8d.

### 4.3. Experimental Method

Histones from calf thymus and 150 bp DNA were used to test the labelling and accumulation in the devices with immobilized dyes. A solution containing histones from calf thymus at a concentration of 2 mg/mL was prepared by reconstituting the lyophilized histone powder in Milli-Q water. The 150 bp DNA was used to be similar to the actual size of cfDNA (147 bp). Different concentrations of 150 bp DNA were made by diluting the 500 µg/mL stock solution. In the case of Qubit immobilization, 2 µL of one-time diluted Qubit was immobilized in the inlet reservoirs in a similar manner to the method mentioned earlier. During the experiment, after the rehydration of agarose gel with 10 µL of 0.2 M CAPS buffer, 1 mg/mL of histones was added to the sample channel. The sample and dried Qubit were mixed by pipetting the sample several times into the inlets. The mixture was allowed to intercalate inside the channel for few minutes before starting the accumulation. 

Human histone H3.1 were used for assessing the dye immobilized device with histones in the presence of BSA sample and human plasma spiked with histone samples. The sample containing histone mixed with BSA was created by diluting the 1 mg/mL stock histone H3.1 solution to 20 and 33 µg/mL and mixed with 40 mg/mL BSA (corresponding to the average HSA concentration in plasma) in 50 mM Tris-HCl buffer pH 8. 

In order to test the device function with dried immobilized GelGreen, 2 µL of 5X GelGreen was immobilized in the reservoirs of sample channel. The 5X GelGreen was created by diluting the 10,000X GelGreen in DI water. Then, the buffer channel was filled with 10 µL of 1X TAE buffer for agarose rehydration. Next, a sample containing 5 µg/mL of 150 bp DNA was added to the sample channel. The sample was pipetted into the reservoirs several times to help with the mixing of DNA and dried GelGreen. The mixture was allowed to sit inside the channel for few minutes before starting the accumulation. Similarly, different dilution ratios for GelGreen (5X, 10X, and 30X) were tested for the samples containing both DNA and histones.

In the case of the integrated device with two gates for DNA and histone detection from plasma samples, 2.5 µL Qubit with no dilution was deposited in one of the reservoirs connected to sample channel in histone accumulation section. Qubit was not diluted as more protein is present in plasma sample (almost doubled the BSA concentration in the previous sample). Also, 2.5 µL of 30X GelGreen was dried in the sample channel’s main inlet and the outlet of the sample channel in the DNA concentration section of the device. Histone and 150 bp DNA spiked in plasma were achieved by spiking histone H3.1 and 150 bp DNA in plasma from healthy persons to obtain final concentrations of 1 and 5 µg/mL of DNA and 20 and 33 µg/mL of histone. For each experiment, 30 µL of the sample was pipetted into the sample channels. The sample and dried dyes were mixed thoroughly by pipetting multiple times into the reservoirs.

### 4.4. Experimental Setup

**Figure 9:**
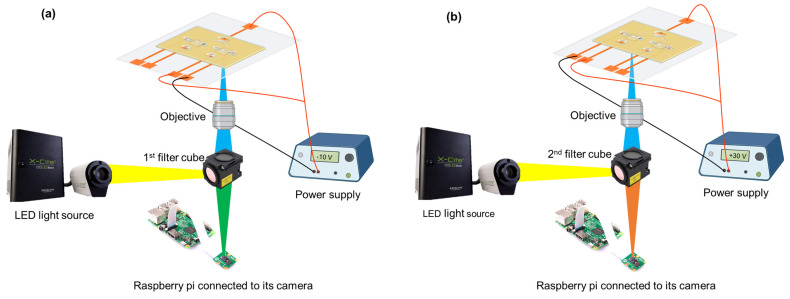
Experimental setup for each part of experiment. (**a**) The setup for DNA detection part. (**b**) The setup for histone detection.

Each experiment was performed using a new device. The microfluidic device was positioned on a fluorescence microscope (Nikon Eclipse TE2000-S, Nikon Instruments Inc. Melville, NY, USA). Two different filter cubes, matching the excitation and emission wavelengths of each dye (GelGreen and Qubit), were used. A filter cube with an excitation window of 472 ± 30 nm and an emission window of 520 ± 35 nm, matching the excitation and emission of GelGreen dye, was used for the DNA accumulation part. Another filter cube with excitation window of 455 ± 25 nm and an emission window of 600 ± 30 nm, according to the excitation and emission of Qubit dye, was used for the histone part of experiment. The light source for the microscope was X-Cite 120LED, and it was operated with 30% of the maximum exposure. The microscope was connected to a raspberry pi camera, programmed to activate the light source one second prior to capturing each image. Images were taken at 30 s intervals, with an exposure time of 4 s and an ISO setting of 400. To minimize interference from external background light, a black cloth covered the device during the imaging process. For each image, the objective was centered on the agarose gates of the device. The experimental setup for DNA and histone accumulation is shown in Figure 9. First, dried agarose gates were rehydrated by filling the top channels with the corresponding buffers. For the first part of the experiment (DNA accumulation), the filter cube suitable for GelGreen dye was utilized. The device electrodes linked to sample and buffer channels for DNA concentration were connected to a power supply (Keithley 2410, Keithley Instruments, Inc. Cleveland, OH, USA). Then, sample channels were filled. After 2 min of waiting for the DNA staining with GelGreen, 10 V was applied between the sample channel under the agarose gate at pH 8.5 and its buffer channel (Figure 9a). Images were taken every 30 s for 5 min. When DNA accumulation was completed, the filter cube was changed to the one matching the Qubit and objective was centered on the agarose gate with pH 11. Electrodes were also connected to the channels of the agarose gate with pH 11 for histone accumulation. After 12 min of waiting time for Qubit and sample protein labelling, 30 V was applied between the channels (Figure 9b). Images were taken every 30 s for 5 min.

### 4.5. Image Analysis

The captured images by the raspberry pi camera through both filters were analyzed using ImageJ. Original images were converted to grayscale. A region of interest (ROI) was defined around the agarose gates, represented by a slightly larger circle encompassing the original gate to account for any misalignment of the adhesive layers and to cover all accumulations in the membrane. After deducting the grayscale value obtained for each image from the initial grayscale value (background), the new value was used for representing the fluorescence intensity at that time point. The background value for each part of the experiments (DNA and histone accumulation) was determined as the average ROI value for all devices at the time when the agarose was completely rehydrated. 

## Data Availability

The data presented in this study are available on request from the corresponding author.

## References

[1] Singer M., Deutschman C.S., Seymour C.W., Shankar-Hari M., Annane D., Bauer M., Bellomo R., Bernard G.R., Chiche J.-D., Coopersmith C.M. (2016). The Third International Consensus Definitions for Sepsis and Septic Shock (Sepsis-3). Jama.

[2] Li Y., Wan D., Luo X., Song T., Wang Y., Yu Q., Jiang L., Liao R., Zhao W., Su B. (2021). Circulating Histones in Sepsis: Potential Outcome Predictors and Therapeutic Targets. Front. Immunol..

[3] McGregor C. (2014). Improving Time to Antibiotics and Implementing the “Sepsis 6”. BMJ Open Qual..

[4] Charoensappakit A., Sae-khow K., Rattanaliam P., Vutthikraivit N., Pecheenbuvan M., Udomkarnjananun S. (2023). Cell-Free DNA as Prognostic and Diagnostic Biomarkers for Adult Sepsis: A Systematic Review and Meta-Analysis. Sci. Rep..

[5] Kim M.-H., Choi J.-H. (2020). An Update on Sepsis Biomarkers. Infect. Chemother..

[6] Cheng Z., Abrams S.T., Alhamdi Y., Toh J., Yu W., Wang G., Toh C.-H. (2019). Circulating Histones Are Major Mediators of Multiple Organ Dysfunction Syndrome in Acute Critical Illnesses. Crit. Care Med..

[7] Dwivedi D.J., Toltl L.J., Swystun L.L., Pogue J., Liaw K.-L., Weitz J.I., Cook D.J., Fox-Robichaud A.E., Liaw P.C. (2012). Prognostic Utility and Characterization of Cell-Free DNA in Patients with Severe Sepsis. Crit. Care.

[8] Eichhorn T., Linsberger I., Lauková L., Tripisciano C., Fendl B., Weiss R., König F., Valicek G., Miestinger G., Hörmann C. (2021). Analysis of Inflammatory Mediator Profiles in Sepsis Patients Reveals That Extracellular Histones Are Strongly Elevated in Nonsurvivors. Mediat. Inflamm..

[9] Jing Q., Leung C.H.C., Wu A.R. (2022). Cell-Free DNA as Biomarker for Sepsis by Integration of Microbial and Host Information. Clin. Chem..

[10] Wildhagen K.C., Wiewel M.A., Schultz M.J., Horn J., Schrijver R., Reutelingsperger C.P., van der Poll T., Nicolaes G.A. (2015). Extracellular Histone H3 Levels Are Inversely Correlated with Antithrombin Levels and Platelet Counts and Are Associated with Mortality in Sepsis Patients. Thromb. Res..

[11] Gould T., Lysov Z., Liaw P. (2015). Extracellular DNA and Histones: Double-edged Swords in Immunothrombosis. J. Thromb. Haemost..

[12] Marsman G., Zeerleder S., Luken B.M. (2016). Extracellular Histones, Cell-Free DNA, or Nucleosomes: Differences in Immunostimulation. Cell Death Dis..

[13] van der Meer A.J., Kroeze A., Hoogendijk A.J., Soussan A.A., van der Schoot C.E., Wuillemin W.A., Voermans C., van der Poll T., Zeerleder S. (2019). Systemic Inflammation Induces Release of Cell-Free DNA from Hematopoietic and Parenchymal Cells in Mice and Humans. Blood Adv..

[14] Zeerleder S., Stephan F., Emonts M., De Kleijn E.D., Esmon C.T., Varadi K., Hack C.E., Hazelzet J.A. (2012). Circulating Nucleosomes and Severity of Illness in Children Suffering from Meningococcal Sepsis Treated with Protein C. Crit. Care Med..

[15] Rhodes A., Wort S.J., Thomas H., Collinson P., Bennett E.D. (2006). Plasma DNA Concentration as a Predictor of Mortality and Sepsis in Critically Ill Patients. Crit. Care.

[16] Gargano A.F., Shaw J.B., Zhou M., Wilkins C.S., Fillmore T.L., Moore R.J., Somsen G.W., Paša-Tolić L. (2018). Increasing the Separation Capacity of Intact Histone Proteoforms Chromatography Coupling Online Weak Cation Exchange-HILIC to Reversed Phase LC UVPD-HRMS. J. Proteome Res..

[17] Kailasa S.K., Kang S.H. (2009). Microchip-Based Capillary Electrophoresis for DNA Analysis in Modern Biotechnology: A Review. Sep. Purif. Rev..

[18] Shahriari S., Selvaganapathy P.R. (2022). Integration of Hydrogels into Microfluidic Devices with Porous Membranes as Scaffolds Enables Their Drying and Reconstitution. Biomicrofluidics.

[19] Shahriari S., Damodara S., Selvaganapathy P.R. (2024). Isoelectric Trapping and Discrimination of Histones from Plasma in a Microfluidic Device Using Dehydrated Isoelectric Gate. Microchim. Acta.

[20] Shahriari S., Patel V., Selvaganapathy P.R. (2023). Xurography as a Tool for the Fabrication of Microfluidic Devices. J. Micromech. Microeng..

